# Measuring empathy in pediatrics: validation of the Visual CARE measure

**DOI:** 10.1186/s12887-018-1050-x

**Published:** 2018-02-13

**Authors:** Michele Arigliani, Luigi Castriotta, Anna Pusiol, Annachiara Titolo, Enrico Petoello, Alberto Brun Peressut, Elisabetta Miorin, Iana Elkina, Federico Marzona, Davide Cucchiaro, Elisa Spanghero, Matteo Pavan, Raffaele Arigliani, Stewart W. Mercer, Paola Cogo

**Affiliations:** 1 Department of Clinical and Experimental Medical Sciences, Unit of Pediatrics, University Hospital of Udine, ASUI Udine, Pediatria, P.zzale S. Maria Misericordia 1, 33100 Udine, Italy; 2 Institute of Hygiene and Clinical Epidemiology, University Hospital of Udine, ASUI Udine, Istituto Igiene, P.zzale S. Maria Misericordia 1, 33100 Udine, Italy; 3 University of Udine School of Medicine, Piazzale M. Kolbe, 3 - 33100 Udine, 33100 Udine, Italy; 4 Department of Surgery, University Hospital of Udine, ASUI Udine, Clinica Chirurgica, P.zzale S. Maria Misericordia 1, 33100 Udine, Italy; 5 University of Trieste, Facoltà di Medicina e Chirurgia dell’Università degli Studi di Trieste, Strada di Fiume, 447, 34149 Trieste, Italy; 6 Pediatric Primary Care, ASL Benevento, Via Giuseppe Piermarini, 12, 82100 Benevento, Italy; 7 General Practice and Primary Care, Institute of Health and Wellbeing, University of Glasgow, 1 Lilybank Gardens, Glasgow, G12 8RZ Scotland

**Keywords:** Not included in the title, Quality of health care, Patient satisfaction, Emergency medical services

## Abstract

**Background:**

Empathy is a key element of “Patient and Family Centered Care”, a clinical approach recommended by the American Academy of Pediatrics. However, there is a lack of validated tools to evaluate paediatrician empathy. This study aimed to validate the Visual CARE Measure, a patient rated questionnaire measuring physician empathy, in the setting of a Pediatric Emergency Department (ED).

**Methods:**

The empathy of physicians working in the Pediatric ED of the University Hospital of Udine, Italy, was assessed using an Italian translation of the Visual Care Measure. This test has three versions suited to different age groups: the 5Q questionnaire was administered to children aged 7–11, the 10Q version to those older than 11, and the 10Q–Parent questionnaire to parents of children younger than 7.

The internal reliability, homogeneity and construct validity of the 5Q and 10Q/10Q–Parent versions of the Visual Care Measure, were separately assessed. The influence of family background on the rating of physician empathy and satisfaction with the clinical encounter was also evaluated.

**Results:**

Seven physicians and 416 children and their parents were included in the study. Internal consistency measured by Cronbach’s alpha was 0.95 for the 10Q/10Q–Parent versions and 0.88 for the 5Q version. The item-total correlation was > 0.75 for each item. An exploratory factor analysis showed that all the items load onto the first factor.

Physicians’ empathy scores correlated with patients’ satisfaction for both the 10Q and 10Q–Parent questionnaires (Spearman’s rho = 0.7189; *p* < 0.001) and for the 5Q questionnaire (Spearman’s rho = 0.5968; *p* < 0,001). Trust in the consulting physician was lower among immigrant parents (OR 0.43. 95% CI 0.20–0.93).

**Conclusions:**

The Visual Care Measure is a reliable second-person test of physician empathy in the setting of a Pediatric Emergency Room. More studies are needed to evaluate the reliability of this instrument in other pediatric settings distinct from the Emergency Room and to further evaluate its utility in measuring the impact of communication and empathy training programmes for healthcare professionals working in pediatrics.

## Background

A “Copernican revolution” is happening in medicine with the shift from the traditional “doctor-centered” approach to a “patient centered” model where healthcare is tailored to the patient’s needs, values and expectations [1–5]. In pediatrics, this approach is embodied in the *Patient and Family Centered Care* (PFCC) programme, which sets out behavioral guidelines and procedures to be practised by healthcare professionals in order to build a real partnership with patients and their families [6, 7].

Empathy is essential to PFCC as it enables the clinician to see the disease “through the patient’s eye” [1, 8, 9]. There is evidence to suggest that an empathetic approach in clinical practice improves patient satisfaction [10, 11] and compliance to medical therapies [12, 13], thus having a positive impact on clinical outcomes [14–16]. A physician’s empathy and communication skills can be improved through a professional training [17–19], a practice that is becoming more widespread in pediatrics [20–22]. Nevertheless, there remains a lack of reliable tools to measure empathy amongst healthcare professionals operating in the pediatric clinical setting [23, 24].

The Consultation and Relational Empathy (CARE) Measure is a patient-rated questionnaire evaluating physician empathy in clinical settings [25, 26]. It has been extensively validated for use in the adult care setting [27–34] and to date seems to be the most reliable second person measure of empathy [23]. A pediatric version of the CARE Measure, the Visual CARE questionnaire, had a preliminary evaluation for use by allied health professionals [35]. This tool investigates the ability of the physician to a) understand the patient’s situation, family circumstances, perspective and feelings; b) communicate effectively with the family; c) act with the patient and his family in a helpful (therapeutic) manner [26].

The purpose of this study was to identify a reliable instrument with which to assess paediatrician empathy in our pediatric emergency department (ED), and highlight aspects of the doctor-patient and doctor-family relationship that require improvement in this setting. We aimed to validate the Visual CARE Measure for use in the pediatric ED and to ascertain whether family background and the severity of a child’s illness have an impact on family perceptions of physician empathy in clinical consultations.

## Methods

This prospective cross-sectional survey evaluated the patient-rated level of empathy of physicians working in the Pediatric ED. The study was approved by the Regional Ethics Committee of Friuli-Venezia Giulia, Italy. Written consent from parents and verbal assent from each child included in the study were obtained.

### Study setting and population

The data were collected between August 2015 and December 2015 at the Pediatric ED of the University Hospital of Udine, Italy, where around 18.000 children per annum are seen. A group of pediatricians and residents in pediatrics, randomly selected among the staff of our Pediatric Division, were involved in the survey. The study population was composed of children aged 7–16 years and the parents/legal guardians of children under the age of 7, who were assessed in the Pediatric ED between 8 AM and 10 PM. During the 6-h shifts of each doctor included in the study, the triage nurse randomly selected patients and, at the end of the consultation, communicated to the consulting doctor that the child and accompanying parent/legal guardian were eligible for the study. In said cases, the doctor invited the patient and family to participate to the survey and, if accepted, a questionnaire was answered by the patient and accompanying parent/legal guardian in the reception area of the Pediatric Emergency Room. Up to 4 patients for each doctor were included in the survey each day. Exclusion criteria were a failure to gain the consent of the parent/legal guardian or the inability of the patient and their parent/s or legal guardian to comprehend/complete the survey instrument (i.e. foreign families with poor Italian language skills).

### Measurements

The survey instrument was a self-administered questionnaire that included the Italian version of the Visual CARE measure and some further questions for the parents.

The Visual CARE measure includes three different questionnaires: (1) 5Q – for children aged 7–11 years; (2) 10Q – for subjects aged 12–16 years; and (3) 10Q–Parent -for parents of children aged 0–6 years or older children unable to complete the questionnaire by themselves. While the 10Q–Parents Visual CARE Measure was answered by parents, the 10Q and 5Q questionnaires were filled in by the patients, who had the possibility to ask for help from their parents if they did not understand the questions. The 10Q and 10Q–Parent questionnaires pose the same questions (10 items as total) and only differ in question wording to reflect who is completing the measure. They were considered as one questionnaire when calculating the sample size and interpreting the results. The 5Q measure has five questions and less complex vocabulary. For all the versions of the Visual CARE Measure, the response options are based on a 5-point faces scale with scores from poor to excellent and a ‘not applicable’ option [35].

The second part of the questionnaire remains the same for each version (10Q, 10Q–Parents and 5Q) and is addressed to parents. In this section, parents were required to provide information on their nationality and education, and to indicate their level of satisfaction with the clinical consultation and the trust in the consulting physician on a scale of 1 to 10 (1 = lowest). In addition, the presence of chronic disease in their child was investigated and the parents expressed the degree of concern for their child’s acute illness on a scale of 1 to 5 where 1 indicated the lowest level of concern. A triage code was interpreted as an indicator of the severity of the disease.

The translation to Italian from the English Visual Care measure was performed in accordance with international standards for the translation and cultural adaptation of patient-reported outcomes measures [36]. A final back-translation into English was performed and this was reviewed by the original developer of the CARE Measure (SWM).

### Statistical analyses

A descriptive analysis of patients’ characteristics and family background was performed. We calculated the proportion of ‘not applicable’ responses for each item (i.e. when patients ticked a ‘not applicable’ box) for each Visual CARE measure (10Q, 10Q–Parent and 5Q). Unanswered items were coded as ‘missing’. Those Visual Care Measures that had either missing values or “not applicable” responses were excluded from the evaluation of the questionnaires’ total score, which was calculated in a range of 10 to 50 for the Visual Care 10Q/10Q–Parent and of 5 to 25 for the 5Q version.

Internal reliability was assessed by Cronbach’s alpha coefficient [37] and according to whether the removal of any of the items weakened Cronbach’s alpha. Homogeneity was evaluated by corrected item-total correlations. Construct validity was assessed by a comparison of the Visual CARE measure scores with overall patient satisfaction.

The sample size for Cronbach’s alpha reliability studies is often calculated as the minimum sample size required for desired lower bound alpha of one-sided confidence interval [38]. In our study, for an expected lower bound Cronbach’s alpha of 0.87 at 95% CI, a minimum sample size of 79 subjects for the 10Q/10Q–Parents questionnaire and 91 children for the 5Q version were needed. The expected lower bound alpha was established by balancing the findings from the validation studies of the adult CARE Measure [27, 28, 34] and those from other studies evaluating clinical scales, that accepted Cronbach’s alpha values ≥0.7–0.8 to prove internal consistency of the test [39–41]. In order to test the internal structure of the 10Q/10Q–Parent Visual CARE measure and evaluate whether any item formed a distinct construct, an exploratory factor analysis was performed [40, 42]. This analysis was conducted by examining the factor loading of each item through principal factor analysis (minimum value of eigenvalues to be retained≥1).

Crude associations between ordinal variables were assessed using the Wilcoxon-Mann-Whitney test and Spearman’s correlation coefficient. Multivariable ordered logistic regression was used to model the effect of socio-demographic factors, severity of the illness and parents’ concern, on the Visual CARE Measure scores.

All analyses were performed using STATA (StataCorp. 2013. Stata Statistical Software: Release 13. College Station, TX: StataCorp LP). An alpha-level of 0.05 was chosen as the guide for significance.

## Results

A total of 490 eligible subjects were invited to participate in the survey but only 416 of these returned the questionnaire (rate of acceptance 85.5%) and were hence included in the analysis. Five percent of the measures (n. 22 out of 416) contained missing items and 4% (n. 19/416) presented at least one “not applicable” response. Complete questionnaires were provided by the parents of 232 children under 7 years of age (55.8% of the total, 10Q–Parents questionnaire), 85 children between 7 and 11 years of age (20.4%, 5Q questionnaire), and 58 subjects aged 12 and older (14%, 10Q questionnaire). Seven doctors (3 pediatricians and 4 residents) were rated in the study. The number of participating patients per doctor ranged from 46 to 76 (median 61). The characteristics of the children and their families are summarized in Table 1.

**Table 1:** Characteristics of the study population

	*n* (%)
Gender	
Male	221 (53.2)
Female	195 (46.8)
Nationality (parent/legal guardian)	
Italian	382 (91.8)
Foreign subjects	34 (8.2)
Level of education (parent/legal guardian)	
University	109 (26, 2)
High school	206 (49.5)
Junior high school	76 (18.5)
Missing information	25 (6)
Triage code	
Red (very critical)	–
Yellow (moderately critical)	13 (3.2)
Green (urgent, not critical)	336 (80.8)
White (not urgent).	40 (9.6)
Missing information	27 (6.4)
Chronic disease	
Yes	15 (3.6)
No	401(96.4)
Complete Visual Care measure for age group (years)	
< 7 (Visual Care measure 10Q–Parents)	232 (55.8)
7–11 years (Visual Care measure 5Q)	85 (20.4)
> 11 (Visual Care measure 10Q)	58 (14)
Incomplete Visual Care measure	
...with missing values	22 (5.2)
...with at least one “not applicable” response	19 (4.5)

The median scores of the VISUAL CARE measures were 47/50 (Interquartile range – IQR = 10) for the 10Q/10Q–Parent version and 24/25 (IQR = 4) for the 5Q questionnaire. There was no statistically significant difference in the patient-rated level of empathy of Pediatricians or residents in pediatrics (Wilcoxon-Mann-Whitney test: z = − 0.564; *p* = 0.573). The distribution of single item scores for the Visual CARE measure are shown respectively in Table 2 for the 10Q/10Q–Parent questionnaire and in Table 3 for the 5Q questionnaire. The parents were, generally, very satisfied with the clinical encounter (median rate 9/10, IQR = 2) and reported a high trust in the consulting physician (median rate 9/10, IQR = 2). Almost 50% (198/408; 48%) of them expressed a moderate degree of concern about their child’s illness (corresponding to a rate of 3, on a scale of 1 to 5).

**Table 2:** Distribution of the Visual CARE Measure 10Q/10Q–PARENTS scores

Items How was the doctor at…	Poor %	Fair %	Good %	Very good %	Excellent %	Does not apply %	Missing %
1. Making you/your child feel at ease? *(being friendly and warm towards you/your child)* N. of answers: 324	–	–	8.6	30	60.8	0.6	0.0
2. Letting you/your child tell your ‘story’ *(giving you time to fully describe things in your own words)* N. of answers: 324	–	0.6	11	30.5	57	0.9	–
3 Really listening? *(paying close attention to what you/your child were saying)* N. of answers: 315	–	0.3	11.4	27.5	60.2	0.6	–
4 Being interested in your child as a whole person? *(asking/knowing relevant details about their life, their situation)* N. of answers: 324	–	2.4	13.8	25.7	56.6	1.2	0.3
5 Fully understanding your concerns? *(communicating that s/he had accurately understood your/your child’s problems)* N. of answers: 324	–	0.9	11.6	33.9	51.8	1.2	0.6
6 Showing care and compassion? *(seeming genuinely concerned)* N. of answers: 317	–	1.5	11.4	32.5	52.5	0.9	1.2
7 Being positive? *(having a positive approach and positive attitude)* N. of answers: 324	–	0.4	8.9	29.7	59.8	0.6	0.6
8 Explaining things clearly? *(fully answering your/your child’s questions, giving you/ your child enough information)* N. of answers: 324	–	0.6	7.7	30.2	60.2	0.6	0.6
9 Helping you to take control? *(exploring with you/ your child what you can do to improve your child’s health yourself)* N. of answers: 324	–	0.9	11.2	34.6	49	3.7	0.6
10 Making a plan of action? *(discussing the options, involving you/ your child as much as you want)* N. of answers: 320	–	1.3	13,8	29.7	49	5.6	0.6

**Table 3:** Distribution of the Visual CARE measure 5Q scores (7–11 years)

Items How was the doctor at…	Poor %	Fair%	Good %	Very good %	Excellent %	Does not apply %	Missing %
1 Making you feel happy and relaxed? (*being friendly and caring and making you feel calm*) N. of answers: 92	–	2.1	10.8	26.3	60.8	–	–
2 Asking questions and letting you talk? *(being interested in you and giving you time to speak)* N. of answers: 92	–	2.1	9.8	25.1	63	–	–
3 Listening and understanding? *(paying attention and knowing the things you find difficult)* N. of answers: 92	–	4.3	4.3	28.4	63	–	–
4 Explaining things? *(answering questions, giving you clear information and instructions)* N. of answers: 89	–	1.1	7.6	22.8	64.2	4.3	–
5. Making a plan? *(encouraging you, talking about what to do next, involving you as much as you want)* N. of answers: 88	–	3.2	5.5	27.1	60.9	3.3	

### Reliability and factor analysis of the Visual CARE measure

The internal consistency of the Visual CARE measured by the Cronbach’s alpha was 0.95 for the 10Q/10Q–Parent and 0.88 for the 5Q version. The removal of any of the items of the questionnaires caused a slight reduction in the Cronbach’s alpha coefficient (Tables 4 and 5). The item-total correlation was > 0.75 for all the items (Tables 4 and 5).

**Table 4:** Reliability, homogeneity and Factor Analysis of the Visual CARE Measure 10Q/10Q–Parents

Items How was the doctor at…	Corrected item-total correlation	Average inter-item covariance	Cronbach’s alpha if item deleted	Factor 1	Uniqueness
1. Making you/your child feel at ease?	0.83	0.36	0.94	0.81	0.33
2. Letting you/your child tell your ‘story’	0.85	0.35	0.94	0.83	0.29
3. Really listening?	0.88	0.34	0.94	0.88	0.22
4. Being interested in your child as a whole person?	0.76	0.35	0.95	0.73	0.46
5. Fully understanding your concerns?	0.88	0.34	0.94	0.87	0.23
6. Showing care and compassion?	0.85	0.34	0.94	0.83	0.30
7. Being positive?	0.83	0.36	0.95	0.82	0.32
8. Explaining things clearly?	0.79	0.36	0.95	0.78	0.38
9. Helping you to take control?	0.79	0.36	0.95	0.85	0.26
10. Making a plan of action?	0.86	0.34	0.94	0.86	0.25

**Table 5:** Reliability, homogeneity and Factor Analysis of the Visual CARE Measure 5Q (7–11 years)

Items How was the doctor at…	Item-total correlation	Average inter-item covariance	Cronbach’s alpha if item deleted	Factor 1	Uniqueness
1. Making you feel happy and relaxed?	0.80	0.35	0.87	0.76	0.42
2. Asking questions and letting you talk?	0.89	0.32	0.84	0.89	0.19
3. Listening and understanding?	0.80	0.35	0.87	0.76	0.40
4. Explaining things?	0.82	0.35	0.85	0.77	0.40
5. Making a plan?	0.81	0.35	0.86	0.75	0.43

The exploratory factor analysis showed that all the items load onto the first factor for both the10Q/10Q–Parent Visual Care Measure (Table 4, Eigenvalue 6.9), and for the 5Q version (Table 5, Eigenvalue 3.1). Uniqueness, which represents the proportion of the variance of the variable that is not associated with the factors [43], was relatively low (Tables 4 and 5). Varimax rotation was not performed as only a single factor was retained. The Visual CARE Measure score significantly correlated with patient satisfaction for both the 10Q/10Q–Parent (Spearman’s rho = 0.72 *p* < 0.001) and the 5Q questionnaire (Spearman’s rho = 0.60; *p* < 0.001). Furthermore, the confidence inspired by the physician was related to his patient rated level of empathy (Spearman’s rho for the 10Q/10Q–Parent questionnaire = 0.69; *p* < 0.001). While foreign parents tended to report a lower trust in the consulting physician (OR 0.43. 95% CI 0.20–0.93), their rating of physician empathy did not significantly differ from that of Italian parents (Wilcoxon-Mann-Whitney tests 10Q/10Q–Parent: z = 1.414; *p* = 0.157. 5Q: z = 0.809; *p* = 0.419). No other significant association was found between the Visual CARE Measure scores and the variables considered, including parents’ level of education, their degree of concern about the child’s illness and the severity of the illness according to the triage code (data not shown).

## Discussion

This study evaluated the reliability of the Visual Care Measure for assessing physicians’ empathy in the setting of Pediatric emergency care.

According to the American Academy of Pediatrics (AAP), when interviewing patients and their families, “there is a need of effectively communicate empathy. Pediatricians also need to recognize the experience, culture, and values and the impact of their personal issues on the therapeutic relationship” [44]. These aspects are embraced in the PFCC initiative [6], which the AAP recommends implementing in the setting of pediatric emergency care [7]. The absence of validated measures of physician empathy in this setting hinders the implementation of the PFCC approach, as physicians cannot receive objective feedback on their strengths and weaknesses regarding their relationships with patients and their families.

In order to address this shortcoming, we tested the Visual CARE Measure, the pediatric version of the most reliable second person measure of empathy [23], in the setting of the Pediatric ED. We also aimed to identify which aspects of doctor-patient relationship most required intervention in our setting and where communication could be improved.

### Validation of the Visual CARE measure

The adult CARE Measure was originally developed in the UK as a measure of physician empathy from the perspective of patients in the adult primary care setting [25, 30].It was subsequently translated into Japanese [27], Croatian [31], Chinese [32] and German [45] versions and evaluated in a range of settings. The CARE measure, which is now widely used internationally, was also shown to be a reliable measure of physician empathy across different specialities in secondary care [33, 34]. The pediatric Visual CARE Measure had been previously evaluated for use by allied health professionals [35]. In keeping with the latter study, we found a high level of internal consistency (the extent to which the items measure the same phenomena) between the 10Q/10Q–Parent questionnaire, as shown by a Cronbach’s alpha coefficient [46] of > 0.90, while the 5Q questionnaire had a lower Cronbach’s alpha in our survey (0.88), albeit higher than previously reported in allied health professionals (0.74) [35]. The lower internal consistency of the 5Q questionnaire may depend on the smaller number of items in the measure [47]. The internal consistency of the Visual CARE 10Q/10Q–Parents questionnaire is comparable to that of the adult CARE measure in primary and secondary care settings [25, 33, 34]. In our study, the elimination of single items had a negligible effect on the Cronbach’s alpha in both the 10Q/10Q–Parent and 5Q Visual CARE Measure, in contrast to the findings of previous studies undertaken in relation to the adult CARE measure [25, 27, 32].

We were able to demonstrate that the items of both the 10Q/10Q–Parent and 5Q questionnaires are satisfactorily homogeneous, as shown by an item-total correlation higher than 0.75 for each item (range 0.76 to 0.89; Tables 4 and 5).

The factor analysis suggested a robust internal structure for the questionnaires, showing that both the versions load onto the first factor (Tables 4 and 5). A previous study in a secondary care setting had similar findings with the adult CARE measure [34]. The rate of incomplete Visual CARE Measures or those with “not applicable” responses (respectively 5% and 4% of the total questionnaires returned) was is in keeping with previous results for the adult version in primary care [27, 32] but slightly higher than in the secondary care setting [34]. The construct validity was supported by a positive strong correlation between the 10Q/10Q–Parent Visual CARE Measure and patients’ overall satisfaction with the consultation (measured on a scale of 1 to 10), and this was consistent with previous evaluations of the CARE Measure [25, 27, 32]. The correlation was moderate for the 5Q–version, suggesting that the construct validity of this version may require further verification using a larger sample.

### Physician empathy and family background

The average Visual Care Measure score for each doctor being evaluated in the survey (44.5/50 for the 10Q/10Q–Parent questionnaire and 22.5/25 for the 5Q version) was reasonably high. Place et al., in their evaluation of the Visual CARE measure for allied-health- professionals, reported similar results, as did the two studies by Mercer et al. testing the adult CARE measure in secondary care settings [33, 34]. In contrast, studies evaluating the empathy of general practitioners reported lower CARE measure scores [25, 27, 31, 32]. There is no clear explanation for the difference in patients’ rating of physician empathy between primary and secondary care, that could mainly depend on differences in consultation length, a factor known to influence the quality of doctor-patient relationships [48–50].

The distribution of the scores from poor to excellent was similar for all the items in the Visual CARE Measure. In the 10Q/10Q–Parent questionnaire, item n.10 about making a plan of action with the patient and family had the lowest score, while in the 5Q questionnaire item n.1 relating to the ability of the doctor to make the patient feel happy and relaxed, obtained the worst scores. Physicians involved in the study received feedback on their strengths and weaknesses with regard to their relationship with patients and families, as indicated in the survey, and will have the opportunity to improve their empathy skills by means of a PFCC training programme that has already started in our Pediatric Division.

Our study did not have any “poor” response rates in the 5-point scale of the Visual CARE measure. While previous studies using the adult CARE measure also reported very low rates of “poor” responses [27, 31], the complete absence of ‘poor’ response ratings in our survey raises concern that parents and children may have been influenced by the emotionality linked to the consultation when they rated their doctors. While this hypothesis could not be verified, the mode of data collection - which involved patients and families filling in the questionnaires directly after the consultation - was the same as that applied in most previous CARE measure validation studies [25, 32, 34].

We had hypothesized that the Visual CARE Measure scores would have discriminated between senior and junior doctors, presuming that more experienced physicians would have been able to communicate more effectively with the patient and his/her family. This hypothesis, which was not evidence based, was not borne out by the present study, which showed no significant statistical difference in the Visual CARE Measure scores of pediatric specialists and residents in pediatrics. This result could be due to a low degree of accuracy of the Visual CARE measure, given that the scores were quite high for all the doctors involved in the study. However, it may also indicate that empathy and communication skills are not automatically acquired with age and clinical experience but always require specific training.

In the setting of pediatric emergency care, factors regarding the situation of patients and their families, such as parents’ nationality, level of education, degree of concern, and even the severity of the child’s illness, did not have an impact on their rating of physicians’ empathy. However, it is notable that trust in the consulting physician tended to be lower among immigrant parents. Previous studies have already identified a higher frequency of distrust in physicians among immigrants and ethnic minorities [51–53]. Our report confirms this data, highlighting the importance of dedicating special attention and resources to the quality of communication with patients who have different ethnic and cultural backgrounds [54, 55].

### Strengths and limitations

One of the strengths of this study is that the translation of the original English Visual CARE Measure into Italian was performed according to current international standards for Patient-Reported Outcomes [36]. A further strong point was that the sample size needed for validation of the 10Q/10Q–Parent questionnaire was largely exceeded.

However, there are also a number of limitations to the study. For the 5Q questionnaire, the sample size fell short of the target by a few subjects (n. 6). A further limitation is that, due to the organization of our ED, the doctors evaluated were those who had invited patients and their families to participate in the study. However, the questionnaires were answered in the absence of the physicians, who had no opportunity to see the responses. Children who answered the 10Q and 5Q Visual CARE Measure had the possibility to request help from their parents in interpreting the questions. Although this may have influenced some of the results, we prioritized the need for the children to fully understand the items in the questionnaires. A total of 74 patients and families refused to take part in the survey or failed to return the questionnaire. We were unable to perform a non-response analysis and are therefore unable to provide information concerning the characteristics of this group and whether this may have biased the results.

## Conclusions

The Visual CARE measure would appear to be a valid and reliable tool to evaluate physician empathy in the setting of Pediatric Emergency Department. Future research should focus on assessing the reliability of the Visual CARE measure in pediatric settings other than Emergency Departments and on evaluating whether this instrument is useful in measuring the impact of professional training on the empathy and communication skills of healthcare professionals working in pediatrics.

Immigrant parents tend to report lower trust in the consulting physician, indicating the need to implement strategies to overcome cultural barriers in the setting of Pediatric emergency care in Italy.

## Abbreviations

AAP: American Academy of Pediatrics
CARE: Consultation and relational empathy
ED: Emergency department
PFCC: Patient and family centered care
